# New Cytogenetic Photomap and Molecular Diagnostics for the Cryptic Species of the Malaria Mosquitoes *Anopheles messeae* and *Anopheles daciae* from Eurasia

**DOI:** 10.3390/insects12090835

**Published:** 2021-09-17

**Authors:** Gleb N. Artemov, Valentina S. Fedorova, Dmitriy A. Karagodin, Ilya I. Brusentsov, Elina M. Baricheva, Igor V. Sharakhov, Mikhail I. Gordeev, Maria V. Sharakhova

**Affiliations:** 1 Laboratory of Evolutionary Genomics of Insects, Institute of Cytology and Genetics, 630090 Novosibirsk, Russia; g-artemov@mail.ru (G.N.A.); karagodin@bionet.nsc.ru (D.A.K.); brusentsovi@gmail.com (I.I.B.); igor@vt.edu (I.V.S.); 2 Department of Genetics and Cell Biology, Tomsk State University, 634050 Tomsk, Russia; klimovavs42@gmail.com; 3 Laboratory of Cell Differentiation Mechanisms, Institute of Cytology and Genetics, 630090 Novosibirsk, Russia; barich@bionet.nsc.ru; 4 Department of Entomology, Virginia Polytechnic Institute and State University, Blacksburg, VA 24061, USA; 5 Department of General Biology and Ecology, Moscow Region State University, 141014 Moscow, Russia; gordeev_mikhail@mail.ru

**Keywords:** mosquito, cytogenetic map, inversion polymorphism, molecular diagnostic

## Abstract

**Simple Summary:**

The most dangerous vectors of malaria in the northern regions of the world belong to the Maculipennis group. Among the 22 species in this group, six are considered dominant vectors of malaria. Of these six, *Anopheles messeae* represents the most widely spread and genetically diverse species in Eurasia and *Anopheles daciae*, a cryptic species whose taxonomic status is still under debate, has been differentiated from *An. messeae* based on differences in their ribosomal DNA. However, genetic studies of these species are scarce. The availability of well-developed polytene chromosomes in malaria mosquitoes provides an opportunity to construct high-resolution cytogenetic photomaps that can be used to investigate the genetic divergence between these species. In this study, we created a standard universal cytogenetic map for the salivary gland polytene chromosomes of *An. messeae* and *An. daciae* and developed a simple and robust molecular approach for species diagnostics. The quality of the cytogenetic map was validated by studying inversion polymorphisms in populations of *An. messeae* and *An. daciae* from a location in the Asian part of Russia. The map will facilitate further investigation of the genetic diversity of these cryptic species.

**Abstract:**

The Eurasian malaria vector *Anopheles messeae* is a widely spread and genetically diverse species. Five widespread polymorphic chromosomal inversions were found in natural populations of this mosquito. A cryptic species, *Anopheles daciae,* was differentiated from *An. messeae* by the presence of several nucleotide substitutions in the Internal Transcribed Spacer 2 (ITS2) region of ribosomal DNA. However, because of the absence of a high-quality reference cytogenetic map, the inversion polymorphisms in *An. daciae* and *An. messeae* remain poorly understood. Moreover, a recently determined heterogeneity in ITS2 in *An. daciae* questioned the accuracy of the previously used Restriction Fragment Length Polymorphism (RFLP) assay for species diagnostics. In this study, a standard-universal cytogenetic map was constructed based on orcein stained images of chromosomes from salivary glands for population studies of the chromosomal inversions that can be used for both *An. messeae* and *An. daciae.* In addition, a new ITS2-RFLP approach for species diagnostics was developed. Both methods were applied to characterize inversion polymorphism in populations of *An. messeae* and *An. daciae* from a single location in Western Siberia in Russia. The analysis demonstrates that cryptic species are remarkably different in their frequencies of chromosomal inversion variants. Our study supports previous observations that *An. messeae* has higher inversion polymorphism in all autosomes than the cryptic species *An. daciae*.

## 1. Introduction

*Anopheles messeae* is one out of six of the dominant malaria vectors in the Eurasian Maculipennis group. It has wide geographical distribution, ranging from the British Islands in the west to the Amur–Zeya Plain in the east and from the city of Salekhard in the north to the Kyrgyz ridge and the Issyk-Kul hollow in the south [1,2,3]. As a result of such a distribution, *An. messeae* is characterized by a high level of genetic diversity [4], including five chromosomal inversions in different chromosome arms [5,6]. These inversions are associated with geographic, ecological, and behavioral adaptations of the species [7,8,9,10,11]. Moreover, a deeper investigation of the inversion frequencies indicated that these inversions were associated unevenly in natural populations and led to the formation of two chromosomal complexes [12]. These chromosomal complexes were referred to as cryptic species *An. messeae* A and B [13]. A later study indicated that the species *An. messeae* A is synonymous to the cryptic species *Anopheles daciae* [14,15,16], which was discriminated from *An. messeae* based on five nucleotide substitutions in the Internal Transcribed Spacer 2 (ITS2) and several morphological differences at the egg stage [17,18]. More recently, the dramatic differences in the frequencies of chromosomal inversions between *An. messeae* and *An. daciae* were shown in three populations from the Moscow region [4]. Moreover, chromosomal variant X1 was fixed in *An. messeae* populations. Based on whole-genome sequencing, this study also discovered genome-wide divergence between these two cryptic species that is especially pronounced on the inversion-rich chromosome X. However, inversion polymorphism remains poorly understood in natural populations of *An. daciae* and requires reevaluation in *An. messeae.*

Investigation of chromosomal polymorphism in natural populations of malaria mosquitoes depends on high-quality cytogenetic maps. The first chromosome map for *An. messeae* [6] was drawn in 1972, but, because of the subjectivity of drawn banding patterns, utilization of this map for population studies was problematic. This map had a similar design to previously developed chromosome maps for North American Maculipennis species [19]. All these maps adapted a nomenclature for mosquito karyotype proposed by K. Rai [20], whereas chromosomes were numbered in order of increasing size with shortest, intermediate, and longest chromosomes being referred to as chromosomes 1, 2 and 3, respectively. Maps for the Maculipennis mosquitoes included five chromosome arms. Chromosome 1, or the sex chromosome X, was represented by one chromosomal arm and both autosomes by two chromosomal arms. Chromosome 1 was labeled as the XL arm in the cytogenetic map of *An. messeae* because the longer arm is not polytenized in this species [21]. Later, the original drawn map of *An. messeae* was employed for the development of a more practical photomap based on film images of orcein stained chromosomes [9]. Unlike other photomaps that were developed at that time for species from the *Anopheles gambiae* complex [22] and *Anopheles stephensi* [23,24], the chromosome images in the *An. messeae* photomap were completely straightened, the five chromosomal arms were subdivided by both numbered and lettered subdivisions, and chromosomal inversions were indicated above the chromosome images by brackets. These features of the map made it extremely convenient for population studies. However, because this map was based on film images of the chromosomes, with contrasting black and white banding patterns and low resolution, its application in field studies was still difficult and required a considerable amount of time and training of personnel in cytogenetics. Current digital imaging technology enabled the creation of “user friendly” high-quality cytogenetic maps for three species from the Maculipennis group: the Eurasian malaria vector *Anopheles atroparvus*, [25]; a major malaria vector in the Middle East, *Anopheles sacharovi* [26]; and the malaria mosquito with the northernmost distribution in Eurasia, *Anopheles beklemishevi* [27]. However, a high-quality cytogenetic map for the most widely spread malaria vector in Eurasia, *An. messeae*, was not developed.

As we mentioned before, the cryptic species *An. daciae* was distinguished from *An. messeae* by the presence of five nucleotide substitutions in ITS2 of its ribosomal DNA [17,18]. However, recent studies determined that three of the first substitutions are heterogeneous in *An. daciae* and only the two latest substitutions are diagnostic for *An. daciae* species identification [4,15,28]. As a result of this heterogeneity, the validity of *An. daciae* as a species was doubted in some studies [29]. Moreover, this observation questioned the accuracy of the species identification, which was determined using differences in the three initial heterogeneous substitutions [14,29,30,31]. Thus, development of a new practical approach that employs Restriction Fragment Length Polymorphism (RFLP), based on species specific diagnostic nucleotides, is very important.

In this study, we developed a standard-universal cytogenetic photomap based on digital images of orcein-stained salivary gland polytene chromosomes that enables fast and accurate identification of the chromosomal inversion variants in natural populations of the cryptic species *An. daciae* and *An. messeae*. In addition, we supplemented the map with five maps of homozygous and heterozygous karyotypes for the five most common chromosomal variants: X1, X2, 2R1, 3R1, and 3L1. The utility of the chromosome maps developed here was validated by an analysis of the chromosomal inversions in populations of *An. daciae* and *An. messeae* from a single location in Russia. Finally, we developed and optimized a new RFLP approach for species diagnostics that utilizes species-specific nucleotides in the ITS2 region of the cryptic species.

## 2. Materials and Methods

Mosquito larvae for the cytogenetic map development were collected from 10 natural populations in the Asian part of Russia (Table S1). For the inversion polymorphism analyses, mosquitoes were collected from the village of Teguldet, located in the Tomsk Region of the Asian (Western Siberia) part of Russia. After collection, larvae were immediately fixed in cold Carnoy’s solution (ethanol: glacial acetic acid, 3:1 by volume) and stored before they were used for chromosome preparations or species identification. Each mosquito larvae were then divided into two parts: thoraxes were fixed in Carnoy’s solution for inversion polymorphism analyses, and abdomens and heads were fixed in 95% ethanol for species identification. All samples were labeled and kept separately at −20 °C.

The cytogenetic map was developed from orcein-stained salivary gland polytene chromosomes of previously identified samples of *An. daciae* and *An. messeae* larvae (Table S1). Approximately 70 of the best chromosome images were selected for map development. For polytene chromosome preparations, larvae were dissected in a drop of cold Carnoy’s (ethanol: acetic acid, 3:1 proportion) solution. Salivary glands were isolated using needles and then a drop of 2% lacto-aceto-orcein, made as 1:1 ratio of lactic acid and 2% orcein in 60% acetic acid, (Fisher Scientific International, Inc., Pittsburgh, PA, USA), was placed on them for 10–15 min on a slide. After that, the glands were transferred into a drop of 45% acetic acid, macerated by needles, covered by a coverslip, and squashed under a piece of filter paper by a needle handle [6]. The coverslips were then glued to the slides using a drop of lacto-aceto-orcein stain placed on each corner of the coverslip. For the chromosome measurements, pictures of nine chromosome spreads were obtained with 40× magnification using an AxioImager A1 microscope equipped with a MRc5 digital camera and AxioVision 4.8.1 software (Carl Zeiss, OPTEC, Novosibirsk, Russia). The measurements were carried out using the tools “Measure” and “Length” in the AxioVision 4.8.1 software. For the map development, pictures of the chromosomes were obtained with 100× magnification. Pictures were edited, cropped, aligned, and trimmed in Adobe Photoshop CS2 [32] and chromosomes were divided into numbered divisions and lettered subdivisions according to traditional nomenclature [9], with several modifications.

For the RFLP molecular approach, PCR products of ITS2 from the rDNA were utilized. Among 65 specimens, 48, 10, and 7 were identified as *An. messeae*, *An. daciae*, and *An. beklemishevi*, respectively. To obtain PCR products of ITS2, small fragments of larval carcasses (a part of an abdomen or head) were pre-washed in 95% ethanol and the dried fragments were then placed in PCR plastic tubes containing 1×PCR-buffer (16 mM (NH_4_)_2_SO_4_; 67 mM Tris-HCl, pH 8.9 at 25 °C; 0.1% Tween-20) with 2.5 mM MgCl_2_, 0.2 mM of each dNTPs (Fisher Scientific International, Inc., Pittsburgh, PA, USA), 0.025 u/μL Taq Polymerase (Biolabmix, Novosibirsk, Russia) and 0.5 µM of each primer. For amplification of the ITS2 region, 5,8S_vdir (5′-TGTGAACTGCAGGACACATG-3′) and 28S (5′-ATGCTTAAATTTAGGGGGTA-3′) rDNA primers modified from [33] were utilized. The PCR reaction was performed according to the previously proposed program [15]. PCR products were analyzed by electrophoresis in 1% agarose gel and TBE buffer, after staining with ethidium bromide. *An. beklemishevi* and *An. messeae*/*An. daciae* were separated by mobility of their PCR products (771 bp and 435 bp, respectively). The PCR-product was used for further RFLP analysis. Approximately 100 ng of PCR-product was added to a mix containing 1×SE-buffer B (SibEnzime, Novosibirsk, Russia) and 1 U of RsaI (SibEnzime, Novosibirsk, Russia). The reaction was kept at 37 °C for 1–2 h until the PCR product was digested. *An. daciae* and *An. messeae* have different numbers of restriction sites for the RsaI endonuclease (3 and 4, respectively). The restriction fragment lengths were 10, 47, 71, and 307 bp for *An. daciae* and 10, 47, 71, 72, and 235 for *An. messeae*. Thus, *An. daciae* and *An. messeae* were detected by the longest restriction fragments of ITS2 (307 bp and 235 bp, respectively) after separation in a 1.5–2% agarose gel. To validate this RFLP approach, 23 samples (11 of *An. messeae*, 7 of *An. daciae*, and 5 of their hybrids) from different geographic locations (Table S2) were sequenced using Sanger sequencing.

For the inversion polymorphism analysis, mosquitoes were identified using the described ITS2-RFLP approach (Table S3). Orcein-stained chromosome preparations were obtained as described above for development of the chromosome map. Identification of chromosomal inversions was performed by comparing the chromosome images obtained by a microscope AxioImager A1 (Carl Zeiss, OPTEC, Novosibirsk, Russia) at 40× and 100× magnification to the aligned standard, and inverted chromosomes on the map developed in this study. The inversion variant nomenclature in the analysis was adopted from elsewhere [9]. The arrangements of a complex inversion in the 3L arm were analyzed using GRIMM software [34], and an intermediate variant of 3L was constructed based on the standard 3L variant, using Adobe Photoshop CS2. The data were summarized in Table S3 and the frequencies of inversions were calculated using Microsoft Excel [35]. A test of the Hardy–Weinberg Equilibrium was performed using an exact test based on Monte Carlo permutations of alleles (the number of replicates for the Monte Carlo procedure was 1000) from the R “pegas” package [36]. The pairwise Fst values [37] between the studied populations were calculated using only autosomal inversions by the BEDASSLE package [38].

## 3. Results

### 3.1. The Standard-Universal Cytogenetic Map for the Cryptic Species Anopheles messeae and Anopheles daciae

**Figure 1:**
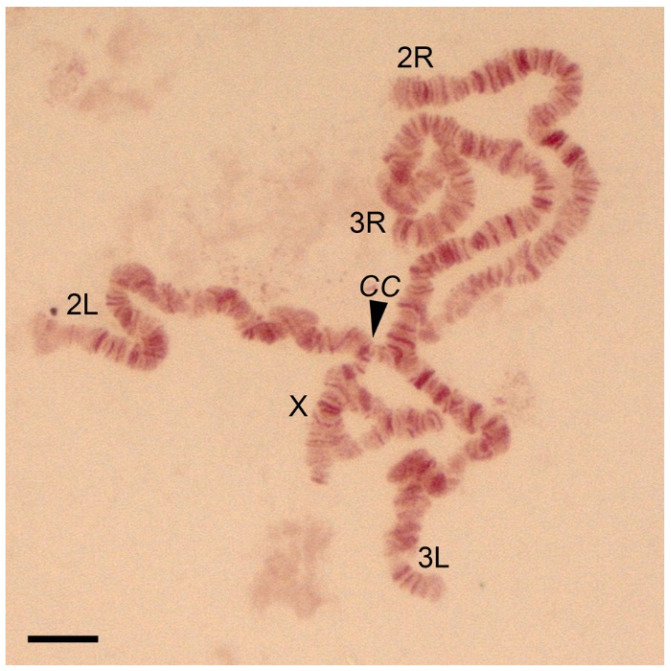
A chromosomal complement of a squashed preparation of salivary gland cells of *Anopheles daciae* with karyotype X01, 2R00, 2L00, 3R01, and 3L00 stained by orcein where X, 2R, 2L, 3R, and 3L represent chromosome armes. Heterozygous inversion variants X01 and 3R01 are seen as loops in the chromosomes. *CC* stands for the chromocenter. Chromosome X and chromosome arms 2R, 2L, 3R, 3L are indicated. Scale bar equals 20 μm.

This study constructed a high-resolution cytogenetic map of the salivary gland polytene chromosomes for the cryptic species *An. messeae* and *An. daciae*. Because no differences in the chromosome banding patterns between these two species have been discovered, except for the inversion frequencies, we consider the map a standard-universal map for *An. messeae* and *An. daciae*. Thus, chromosome images of both species were combined and used for the construction of the map (Table S1). The species were identified using a newly developed ITS2-RFLP approach. A diploid chromosome complement in the malaria mosquitoes *An. messeae* and *An. daciae* consists of six chromosomes, which appear as five chromosome arms in salivary glands because of homologous chromosome pairing [39] (Figure 1). The X sex chromosome is the shortest of the chromosome complement and is represented by only one polytenized arm (Table 1); the other arm is not polytenized [6]. Chromosomes 2 and 3 are almost identical in length. Chromosome 2 consists of two arms of approximately equal lengths. Chromosome 3 has arms of different lengths with 3R being the longest (Table 1). In salivary glands, polytene chromosomes are connected to each other by pericentromeric regions forming a chromocenter (Figure 1). During squashing, the connection between chromosomes often becomes disrupted and chromosome arms separate from each other in the chromosome spread on a slide.

**Figure 2:**
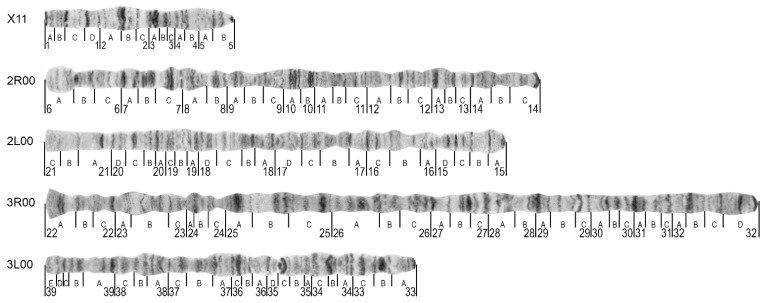
A standard-universal photomap for the malaria mosquitoes *Anopheles messeae* and *Anopheles daciae.* The telomere and centromere ends are shown at the left side and at the right sides of the map, respectively. Chromosome X and chromosome arms 2R, 2L, 3R, and 3L are indicated on the right. The numbered divisions (1–39) and lettered subdivisions (A–E) are shown below the chromosomes.

For development of the standard-universal cytogenetic map for *An. messeae* and *An. daciae*, a karyotypes X11, 2R00, 3R00, 2L00, and 3L00 were used because the X00 variant was absent [4] or only found at low frequencies [40] in natural populations of *An. messeae* (Figure 2). The high-resolution image of this map is shown in Figure S1A. This karyotype was traditionally employed for previous chromosome maps of *An. messeae* [6,9,41] with the exception of 3R, for which the variant 3R11 was utilized. Before placing chromosomal images to the map, the curved parts of the original images were straightened and combined with each other into the whole-arm structure as described previously [32,42]. On the final map, straightened arms are positioned starting with chromosome X on the top and ending with the 3L arm at the bottom (Figure 2). Telomere and centromere ends are oriented to the left and the right sides of the map, respectively. The boundaries between numbered and lettered subdivisions are placed at the band-interband borders following previous nomenclature rules with some modifications [9]. The borders of the divisions and subdivisions in the 3R arm are slightly optimized according to the banding patterns. The numbers of divisions begin from the left in chromosome X and in the 2R and 3R arms, and are in the opposite order, from the right, in the 2L and 3L arms.

**Figure 3:**
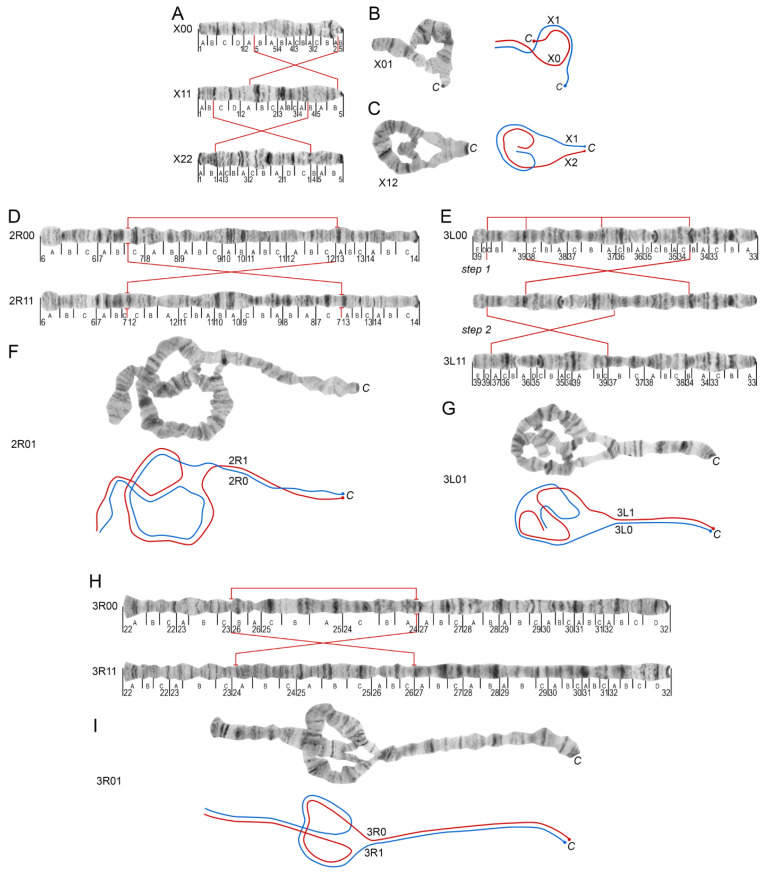
Maps for the chromosomal inversions in *Anopheles messeae* and *Anopheles daciae.* Maps for homokaryotypes X00, X11, X22 in chromosome X are shown in panel (**A**). The numbered divisions (1–39) and lettered subdivisions (A–E) are shown below the straight chromosome images. Straight red lines indicate the cytogenetic positions of inversion breakpoints and their connections between standard and inverted karyotypes. Curved red and blue lines indicate a schematic representation of standard and inverted arrangements, respectively, tracing the axes of homologues. The letter *C* stands for a centromere end. Heterokaryotypes X01 and X12 are shown in panel (**B**,**C**), respectively. Chromosome maps for the 2R arm for homokaryotypes 2R00 and 2R11 are shown in panel (**D**). Chromosome maps of the inversions in the 3L arm of *An. daciae* and *An. messeae* for homokaryotypes 3L00 and 3L11 are shown in panel (**E**). The 3L1 arrangement is expected to originate through two steps, which are shown by crossed red lines in “step 1” and “step 2” (panel (**E**)). An intermediate chromosome variant, created in step 1, has never been found in nature and was assembled by the graphic editor. Panel (**F)** demonstrates a photographic image of the 2R01 heterokaryotype and its schematic representation. Panel (**G**) demonstrates a photographic image of the 3L01 heterozygous variant and its schematic representation. Finally, chromosome maps of the 3R11 inversion of *An. messeae* and *An. daciae* are shown as homozygotes 3R00 and 3R11 in panel (**H**) and as the heterozygous variant 3R01 in panel (**I**).

The most prominent landmarks for chromosome arm identification are described in Table 2. Telomere ends of the chromosome usually have very specific and easily recognizable structures for chromosome arm identification. A specific thin dark band located at the beginning of region 1A can serve as a telomere landmark of chromosome X. Two additional dark bands located in regions 1C, 1D, the puffed region following the dark double band in the 2A,B division, and the light area with diffused bands in divisions 4B–5A are indicative of the X chromosome as well as for the X1 and X2 chromosome inversion arrangements (Figure 3A–C). The 2R arm often has a round-shaped telomere end with wide pale bands followed by a dark band in division 6B (Figure 2). Two sets of dark bands in the middle of the arm in divisions 10A,B can be used as additional landmarks for both arms and inversion diagnostics (Figure 3D). The 2L chromosome arm is easy to distinguish by its long, light end followed by a thin band in division 21A. A short-flared telomere end with two dark thin bands in division 22A is indicative of the 3R arm, and the arrangement of a narrow neck-like region in 24C followed by three sets of several dark bands in divisions 25A, 25B, and 25C can be utilized as additional landmarks. A set of five bands in regions 39B–E and a “bird’s eye”, a dot-like band coupled with long and dark, often curved, bands in region 35C are convenient structures for recognition of the 3L arm. Unlike in the ovaries [25,26,27], centromere ends of the chromosomes in the salivary glands have similar morphology and cannot be used as landmarks.

The nomenclature of the inversions follows a previous study [9,43]. In this study, we included the X1, X2, 2R1, 3R1, and 3L1 chromosomal inversion variants (Figure 3 and Figure S1B). These variants are recognized by the changed positions of the landmarks (Table 2). Heterozygous inversion karyotypes (Figure 3B,C,F,G,I) can be easily distinguished by specific loop-like structures in the chromosomes because the homologous polytene chromosomes are usually paired in salivary glands [44]. The position of the loop in the arm indicates the heterozygote inversion variants, which are the most prominent in short chromosomes like X (Figure 3B,C). In chromosome X, the X0 arrangement exchanges the puffed region in 2B to the light area of the 4B–5A divisions compared to the X1 arrangement (Figure 3A). Banding in pericentric regions in homologous chromosomes and the length of the telomere portion of the X chromosome, which is outside the inversion, are also indicative of inversion variants. In the heterokaryotype X01, the region following the telomere in the chromosome, which is not incorporated into the loop, comprises almost half of the chromosome (Figure 3B). The X2 inversion is based on the original variant X11. This inversion transfers the bands from the 1D and 2B division to the right side of the chromosome, while the puffed region in 2B moves to the left side of the chromosome (Figure 3A,C). Very often homologues are asynaptic in heterozygous X chromosomes and the easiest way to distinguish X01 and X12 is to compare pericentric regions in both homologues. As the X1 arrangement moves the puffed region of 2B near the centromere, the X1 and X0 homologues are remarkably different in the pericentric region, which is never seen in X12. An additional feature of the X12 heterokaryotype is that the non-inverted part of the telomere part of the chromosome is much shorter (about 1/8 part of the chromosome length) than that in X01 (Figure 3C).

The 2R1 inversion flips two sets of dark bands in division 10A,B (Figure 3D), while the 3R1 inversion inverts the band arrangement in the 24B–25C divisions (Figure 3H). Both heterozygous variants 2R01 and 3R01 represent simple inversion loops within the border of inversions (Figure 3F,I). The size and position of the loops in the 2R and 3R chromosome arms are indicative of the inversion variant composition even more than homozygotes 2R11 and 3R11. In the 2R01 variant (Figure 3F), the loop is bigger than in 3R01 and localizes in the middle of the chromosome. In the 3R01 variant (Figure 3I), the loop is smaller than in the 2R01 variant and localizes closer to the telomere end of the chromosome. The 3L1 double inversion transfers the “bird’s eye” landmark in region 35C from the middle part of the arm to the telomere end (Figure 3E). In the heterokaryotype 3L01, the chromosome forms a double loop structure that includes almost the entire chromosome arm (Figure 3G). We performed GRIMM analysis to reconstruct the order of the rearrangements, which resulted in the inverted 3L11 karyotype [34]. The analysis showed that the 3L1 inversion originates from two overlapping inversions (Figure 3E). In the first step, an inversion rearranges the central part of the arm and, in the second step, another inversion in the telomere half of the arm turns it around again. The intermediate variant, which is shown in the middle of Figure 3E and is reconstructed based on the 3L0 arrangement, has never been found in nature. Thus, we hypothesize that the 3L11 homozygote inversion variant is a result of two rearrangements that occur in a short period of time.

### 3.2. Chromosome Map Validation by the Analysis of Inversion Polymorphism in a Single Asian Location

**Figure 4:**
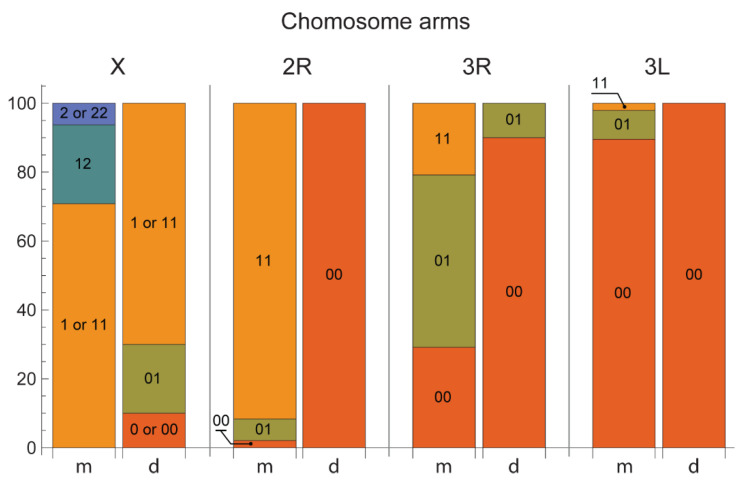
Frequencies of the chromosomal inversions in natural populations of malaria mosquitoes *Anopheles daciae* and *Anopheles messeae* in Teguldet, Western Siberia, Russia. The letters “m” and “d” stand for *An. messeae* and *An. daciae*, respectively. Frequencies of the standard and inverted variants are shown by different colors in chromosome X, and chromosome arms 2R, 3R, and 3L. Homozygous karyotypes are shown as 00, 11, and 22 and heterozygous karyotypes are shown as 01, 02, and 12. Hemizygous karyotypes in males that have only one chromosome X are indicated as 0, 1, or 2.

The utility of the chromosome map for inversion identification analysis was tested on mosquitoes collected from natural populations in Teguldet, located in the Tomsk region of Western Siberia in Russia (Figure 4). The species were identified using the ITS-RFLP approach. Among the cryptic species *An. messeae* and *An. daciae*, five chromosomal inversion variants were observed: X1, X2, 2R1, 3R1, and 3L1. The frequencies of these chromosomal inversions are shown in Table 3. Karyotypes X01, X00, and the hemizygote male specific karyotype X0 were not observed in *An. messeae,* whereas combinations of X12, X22, and X2 were absent in *An. daciae*. A heterokaryotype X02 was not observed in either of the two species. Thus, chromosomal arrangements X0 and X2 were species-specific in this location for *An. daciae* and *An. messeae*, respectively. The frequency of the X1 arrangement was higher than X0 in *An. daciae* (77.8% vs. 22.2%) and the frequency of the chromosomal variant X2 was lower than X1 in *An. messeae* (18.5% vs. 81.5%). The frequencies of the autosomal inversion variants were also very different between species. The inversion variants 2R1 and 3L1 were found only in the *An. messeae* population with high (94.8%) and low (6.3%) frequencies, respectively. The 2R01 and 3L01 karyotypes were found only in *An. messeae*, whereas the 2R00 and 3L00 karyotypes were found in *An. daciae* in the Teguldet population. Inversion 3R1 was found in high and low frequencies in the *An. messeae* and *An. daciae* populations, respectively (46% vs. 5%).

Analysis of population differentiation [37] revealed a highly significant interspecies differentiation Fst = 0.629 (Table 4). However, when checking the Hardy-Weinberg equilibrium [36] there were no deviations from equilibrium within the species on any chromosome (Table 4). Thus, our study demonstrates the high level of genetic divergence between the cryptic species and clearly suggests that cryptic species do not freely reproduce in the Teguldet location.

### 3.3. A New ITS2-RFLP Approach for Anopheles messeae and Anopheles daciae Identification

**Figure 5:**
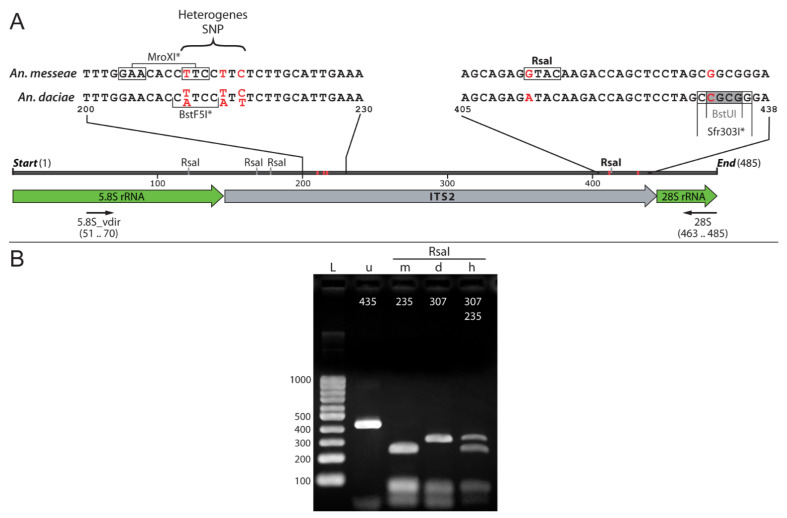
The ITS2-RFLP approach based on the RsaI restriction enzyme for diagnostics of *An. measeae* and *An. daciae*. Panel (**A**) represents a diagram illustrating the structure of ITS2 in *An. messeae* and *An. daciae* and its restriction by enzyme RsaI. Letters on the top indicate sequences of SNP regions and sites for restriction enzymes RsaI, MroXI, BstF5I, BstUI and Sfr303I that were used in different studies for species identification. The structure of ITS2 is shown based on coordinates according to the AY648982 sequence [17]. The positions of five nucleotides that differentiate species are shown by red letters or red stripes. Unique restriction sites are marked by an asterisk. The RsaI restriction site, which was utilized for species diagnostics in this study, is shown in bold. Positions of primers used for PCR amplification are shown by black arrows. Panel (**B**) shows a pattern of ITS2-RFLP restriction by RsaI of PCR products obtained from *An. messeae* (m), *An. daciae* (d), and their hybrid (h). Undigested PCR-product (u) is shown next to the DNA ladder (L).

In this study, a new ITS2-RFLP approach for identification of the cryptic species *An. messeae* and *An. daciae* was proposed. The structure of ITS2 and the surrounding rDNA region is shown in Figure 5. The highly conserved 5.8S and 28S genes are separated by a variable ITS2. Universal primers that produce species-specific PCR products of ITS2 were designed based on rDNA genes. Among five originally described nucleotide substitutions [17], only 2 substitutions in positions 412 and 432 could be used for unambiguous identification of the species, since the first three substitutions in positions 211, 215, 217 are heterogeneous in *An. daciae* [4,31] (Figure 5A). The numbers of the nucleotide positions were given in correspondence to the original *An. messeae* sequence AY648982 [17]. Analysis of the ITS2 sequences allowed development of a new version of species identification using digestions of ITS2-PCR products by the RsaI restriction enzyme. This restriction enzyme cleaves ITS2 of *An. messeae* in 4 places and *An. daciae* in three places (Figure 5A). As a result, the lengths of the fragments after restriction were 10, 47, 71, 72, and 235 nucleotides in *An. messeae*; and 10, 47, 71, and 307 nucleotides in *An. daciae* (Figure 5B). The easiest way to identify the species is by the longest bands in the series: 235 bp or 307 bp. In this study, the method was also validated using previously sequenced ITS2 DNA samples from different populations (Table S2). A total of 23 or 11 of *An. messeae*, 7 of *An. daciae*, and five of their hybrids were digested by RsaI. The species and their hybrids were identified utilizing Sanger sequencing by the presence of GG or AC nucleotides in positions 412 and 432 in *An. messeae* and *An. daciae*, respectively, and by double nucleotides for their hybrids. All samples indicated the correct patterns of ITS2-RFLP bands in agarose gel: one large fragment each for *An. messeae* and *An. daciae* (235 bp and 307 bp, respectively) and both fragments for hybrids.

## 4. Discussion

In this study, a standard-universal cytogenetic photomap was developed for the dominant malaria vector *An. messeae* [1] and a cryptic species *An. daciae* [17]. This map was based on straightened high-quality digital chromosome images and, because no differences were observed in chromosomal banding patterns between the species, it can be universally used for chromosomal studies in both species. Although slightly different chromosome nomenclatures were proposed in the past for *An. messeae* [6,9,41], in this study, we used a nomenclature where the chromosomes were named as the sex chromosome X and autosomes 2 and 3 in order of increasing size. Chromosome Y has low levels of polytenization [45] and is not included in the current map. In multiple studies, the polytenized arm of chromosome X was labeled as XL in *An. messeae* because only the short chromosome arm is polytenized in the salivary glands. Here, we adapted a nomenclature that is used for other mosquitoes including more recent maps for *An. atroparvus* [25] and *An. sacharovi* [26]. According to the first photomap of *An. messeae*, chromosomes in the current map were divided into 39 numbered divisions [9]. The number of 121 lettered subdivisions was smaller for two regions because of optimization of the subdivisions in the 3R arm. Here, we followed the originally proposed rules for cytogenetic maps of *Drosophila* where the chromosome regions begin from the band-interband border and include at least one band [44]. Thus, in the 3R arm, the regions 27 and 29 in the new map were divided into three subdivisions instead of four in the previous map [9]. Similar to the recently developed cytogenetic maps for species from the Maculipennis group [25,26,27], we changed the order of the letter subdivisions in the left arms of chromosomes 2 and 3 from right to left to make this map more convenient for future genome mapping purposes [46].

Unlike other recently developed cytogenetic maps based on phase-contrast ovarian chromosome images [25,26,27,42,47], the current photomap of *An. messeae* and *An. daciae* utilized orcein-stained chromosome images from salivary glands. Orcein stained chromosomes from the salivary glands were commonly used for population studies of inversion polymorphism [5]. Thus, we employed such chromosome images for map development to make it more convenient for population genetic studies in the field. Although different nomenclatures for the chromosomal inversions in *An. messeae* currently exist [16], we followed the most popular chromosome nomenclature, introduced by V.N. Stegniy in 1983 [43], which has been used in a significant number of studies [5,7,8,11,48,49]. To simplify application of the map for population studies, we used chromosome images for the 2R00, 2L00, 3R00, 3L00 karyotypes of the autosomal chromosome arms, and the homozygote variant X11 for the sex chromosome, which was previously included in standard chromosome maps [6,9,41]. Also, the karyotype X11 was used because, according to recent studies, the X0 arrangement is extremely rare [40] or absent in *An. messeae* populations [4,16]. The X11 karyotype was used for all previously constructed chromosome maps for *An. messeae* [6,9,41]. However, a modification of the map for *An. daciae* with the X00 karyptype can be useful for studying European populations where this karyotype is the most common [50]. In addition to the standard-universal map, our study included a detailed description of the chromosomal landmarks for the recognition of the chromosomal variants X1, X2, 2R1, 3R1, and 3L1 and of the structure of inversion homozygotes and heterozygotes. Thus, the cytogenetic map constructed here is convenient for the fast and accurate identification of chromosomal inversions in natural populations of both cryptic species, *An. messeae* and *An. daciae.*

Inversion polymorphism is a well-studied phenomenon in *An. messeae* [5,8,49]. Chromosomal inversions in European populations of this species are associated with certain landscape-climatic zones [51]. However, studies of inversion polymorphism in the cryptic species *An. daciae* are incomplete [4]. Moreover, identification of *An. daciae* as a separate taxon from *An. messeae* requires further revision of inversion polymorphism in *An. messeae*. This became extremely important because the existence of such a taxonomic unit as *An. daciae* was doubted by some researchers [29,52]. Thus, the availability of a high-quality cytogenetic map will help to better understand the genetic diversification of cryptic species. In this study, we validated the quality of our map by an inversion polymorphism analysis in the natural population from Teguldet location in the Tomsk region in Western Siberia in Russia. Indeed, because chromosomal banding patterns were represented on the map in detail, the map simplified analysis of the chromosomes and made recognition of the inversions easy. Similar to a previous study conducted in three Moscow populations [4], our analysis indicated significant differences in inversion frequencies between the cryptic species. Karyotype X00 was completely absent in *An. messeae* populations in European (Moscow) and Asian (Tomsk, Teguldet) locations. However, the inversion frequencies were different between the locations. Arrangement X1 was more abundant in the *An. daciae* population in Europe than in the Asian population, whereas arrangement X2 was found in low frequencies only in the Asian populations of *An. messeae*. Inversion variant 2R1 was more abundant in *An. messeae* in European populations than in Asian populations and absent, or almost absent, in Asian and European populations of *An. daciae*, respectively. In contrast, the 3R1 variant was more abundant in the Asian population than in the European population of *An. messeae* but was present in low frequencies in *An. daciae* in both locations. In both study locations, inversion 3L1 was present in low frequencies only in the *An. messeae* populations. Thus, although the previously observed geographic component [5] in the inversion frequencies was evident, the study suggests that there is high genetic divergence between the species. Overall, *An. messeae* has higher inversion polymorphism than the cryptic species *An. daciae*. These observations suggest that *An. messeae* and *An. daciae* may represent different evolutionary entities with overlapped inversional polymorphism.

Finally, we developed a new ITS2-RFLP approach for *An. messeae* and *An. daciae* identification. ITS2 is one of the most popular neutral markers for species identification in mosquitoes from the *Anopheles* genus [53]. This method was successfully applied for species identification in different *Anopheles* groups or complexes [54,55,56,57]. Moreover, ITS2 sequencing was employed for the discovery of eight new species in the Maculipennis group: *An. hermsi* [58], four new species of the Quadrimaculatus complex [59], *An. artemievi* [60], *An. persiensis* [61], and, finally, *An. daciae* [17]. Most of the species among the Eurasian Maculipennis group can be easily recognized based on the sizes of PCR products and do not require repeated sequence comparison [62,63,64]. However, in some cases, despite the sequence differences, the total size of the ITS2-PCR products is very similar among the different species. For example, the size of the *An. punctipennis* sequence is very similar to other species, such as *An. quadrimaculatus* and *An. crucians* D, which can be present in the same location [65]. Similarly, the cryptic species *An. daciae* was discriminated from *An. messeae* by the presence of five nucleotide substitutions, but the total size of the PCR product remained the same. In such cases, the easiest way to determine differences between the species is to perform an additional digestion using restriction enzymes that cleave the site with different nucleotide arrangements (Figure 5). Originally, five nucleotide substitutions were described for this cryptic species identification [17]. However, later studies discovered that the first three substitutions are heterogenic and can only be utilized for species identification with caution, since *An. daciae* is heterogeneous and contains all variants in the first three substitution positions, which are also present in *An. messeae* [4,15,28]. Thus, the use of the first three substitutions do not allow one to separate the species and their hybrids from each other. In the case of using restriction enzymes that cleave the TTC variant, such as MroXI, which is characteristic of *An. messeae* but is also present in the heterogeneous substitutions of *An. daciae* (Figure 5), it is impossible to separate *An. messeae* from *An. daciae* or hybrids by RFLP, resulting in the misjudgment of the status of the species by these authors [29]. The use of a restriction enzyme, which cleaves the AAT variant, for example, BstF5I [14,31], does not allow separation of *An. daciae* from hybrids, but it is possible, in some cases, to separate *An. daciae* from *An. messeae.* The ITS2-RFLP approach developed here is based on endonuclease RsaI, which cleaves the ITS2 fragment in a unique substitution between *An. daciae* and *An. messeae* at position 412 (Figure 5). It is better in comparison with enzymes, which recognize the first nucleotide from three heterogeneous nucleotide substitutions between both species and their hybrids. Moreover, RsaI has a better restriction patterns than the previously proposed restriction enzymes BstUI [18] or Sfr303I, which utilize a substitution at position 432. Compared to BstUI, with lengths of diagnostic fragments 109 and 59, 52 bp, RsaI produces fragments of 307 and 235 bp that are easier to detect by size. An advantage over Sfr303I is the presence of several sites in ITS2, which makes it possible to reliably detect incomplete hydrolysis. This approach also allows for reduction in the time and cost of species identification and can be utilized for quick diagnostics of field-collected mosquitoes.

## 5. Conclusions

This study constructed a high-quality cytogenetic photomap for two malaria vectors, *An. messeae* and *An. daciae,* which are still considered as cryptic species with unclear taxonomic status [66]. The application of this map for the Asian populations of *An. messeae* and *An. daciae* demonstrated a high level of genetic divergence between the cryptic species. In addition, a simple and robust ITS2-RFLP technique for these species’ identification was developed. These new cytogenetic and molecular tools can be further utilized for studying the taxonomy, systematics, and population genetics of these mosquitoes, which help in developing robust and accurate strategies for vector control.

## Figures and Tables

**Table 1:** Measurements of the polytene chromosomes from *An. daciae* salivary gland cells.

Chromosomes	X	2	3
Average length (μm)	58.5 ± 6.1	297.5 ± 40.1	312.9 ± 36.9
Relative length (%)	8.6	44.5	46.9
Relative short arm length (%)	NA	46.9	38.5

**Table 2:** Important chromosome landmarks for arm and inversion identification.

Chromosome or Chromosome Arms	Telomere	Additional Landmarks
X	Sharp end with the thin dark band in 1A	Puffed region in 2B division starting with a dark band, and the light area with diffused bands in divisions 4B–5A
2R	Often round shape end with wide pale bands followed by a dark band in the 6B division	Two sets of dark bands in the middle of the arm in the 10A,B division
2L	Long, light end limited by a thin band in the 21A division	No additional landmarks identified
3R	Short flared end with two thin bands in division 22A	A neck-like region in 24C followed by 3 sets of several dark bands in the 25A, 25B, and 25C divisions
3L	A set of five bands in 39B–E	A “bird’s eye” landmark in the 35C region—dot-like band coupled with long and dark often curved bands

**Table 3:** Frequencies of the chromosomal variants in populations from Teguldet.

Chromosomal Variant	*An. messeae*	*An. daciae*
X1	81.5%	77.8%
X2	18.5%	0%
2R1	94.8%	0%
3R1	45.8%	5%
3L1	6.3%	0%

**Table 4:** Results of the exact test for Hardy-Weinberg Equilibrium (HWE) and analysis of population differentiation (Fst).

Chromosomes	HWE, *p*-Value *An. messeae*	HWE, *p*-Value *An. daciae*	Fst
X	-	-	0.091
2R	0.104	1.000	0.915
3R	1.000	1.000	0.261
3L	0.153	1.000	0.000
Total	-	-	0.629

## Data Availability

Not applicable.

## Supplementary Materials

The following are available online at https://www.mdpi.com/article/10.3390/insects12090835/s1, Figure S1: High resolution image of a standard-universal photomap (A) and the chromosomal inversions (B) in the malaria mosquitoes *Anopheles messeae* and *Anopheles daciae*. Table S1: Mosquito collections used for the development of the standard-universal chromosome map and illustration of the inversion variant composition. Species were identified by the PCR-RFLP approach using the RsaI endonuclease. Table S2. Genotyping results for specimens based on ITS2 sequences. Letters in sample numbers indicate their locations: IR—Irkutsk; KZ—Kazakhstan, Semey; LR—Latvia, Riga; NB or NB(V)—Novosibirsk, Berdsk (collection “V”); T(B) or T(D)—Teguldet, pond B or D. The presence of double picks in ITS2 sequences (SNP) are indicated by the IUPAC nucleotide ambiguity code as letters M(A+C), R(A+G), S(C+G), W(A+T), and Y(C+T). Table S3. Karyotyping of the specimens based on RFLP analysis of ITS2 sequence.
