# Supplementary material for: New Cytogenetic Photomap and Molecular Diagnostics for the Cryptic Species of the Malaria Mosquitoes Anopheles messeae and Anopheles daciae from Eurasia
Source: Insects. 2021 Sep 17;12(9):835. doi: 10.3390/insects12090835 (PMC8465136; doi:10.3390/insects12090835)
Supplement: Supplementary file 1 [file insects-12-00835-s001.zip › insects-1328149-supplementary.pdf]

Table S1. Mosquito collections used for the development of the standard-universal chromosome map and illustration of the inversion variant composition. Species were identified by the PCR-RFLP approach using the RsaI endonuclease.

**Chromosomal variant**	**Species**	**Populations**
X00	*An. daciae*	Dzerzhinskoye, 08/14/2018
X01	*An. daciae*	Kansk 07/11/2019
X11	*An. daciae,*<br>*An. messeae*	Barnaul, 06/09/2019,<br>Krivosheino, 07/18/2018
X12	*An. messeae*	Barnaul, 06/09/2019
X22	*An. messeae*	Semipalatinsk, 08/22/2017
2R00	*An. daciae*	Kolarovo, 07/03/18
2R01	*An. messeae*	Barnaul, 06/09/2019
2R11	*An. messeae*	Krivosheino, 07/18/2018<br>Teguldet, 08/22/18
3R00	*An. daciae*	Barnaul, 06/09/2019,<br>Kolarovo, 07/03/2018,
3R01	*An. daciae,*<br>*An. messeae*	Dzerzhinskoye, 08/14/2018,<br>Krivosheino, 07/18/2018
3R11	*An. messeae*	Barnaul, 06/09/2018,<br>Zelenogorsk, 07/17/2018,<br>Kansk 07/11/2019,<br>Krivosheino, 07/18/2018
3L00	*An. daciae*	Dzerzhinskoye, 08/14/2018,<br>Kolarovo, 07/03/2018
3L01	*An. messeae*	Barnaul, 06/09/2019
3L11	*An. messeae*	Dzerzhinskoye, 08/14/2018,<br>Kargasok, 07/18/2019,<br>Noyabrsk, 07/27/2019

Table S2. Genotyping results for specimens based on ITS2 sequences. Letters in sample numbers indicate their locations: IR—Irkutsk; KZ—Kazakhstan, Semey; LR—Latvia, Riga; NB or NB(V)—Novosibirsk, Berdsk (collection "V"); T(B) or T(D)—Teguldet, pond B or D. The presence of double picks in ITS2 sequences (SNP) are indicated by the IUPAC nucleotide ambiguity code as letters M(A+C), R(A+G), S(C+G), W(A+T), and Y (C+T).

**Sample number**	**Accession number**	**Species**	**SNP**					
			**150**	**211**	**215**	**217**	**412**	**432**
IR3	MZ578242	*An. messeae*	M	T	T	C	G	G
IR4	MZ578243	*An. messeae*	M	T	T	C	G	G
KZ18	MZ578244	Hybrid	M	T	T	C	R	S
KZ3	MZ578245	*An. messeae*	M	T	T	C	G	G
KZ4	MZ578246	*An. messeae*	M	T	T	C	G	G
KZ6	MZ578247	Hybrid	M	T	T	C	R	S

LR11	MZ578248	*An. daciae*	C	A	W	Y	A	C
LR12	MZ578249	*An. messeae*	M	T	T	C	G	G
LR17	MZ578250	*An. daciae*	C	W	W	Y	A	C
LR2	MZ578251	*An. daciae*	C	W	W	Y	A	C
LR8	MZ578252	*An. daciae*	C	W	W	Y	A	C
LR9	MZ578253	*An. messeae*	M	T	T	C	G	G
NB(V)17	MZ578254	Hybrid	C	T	T	Y	R	S
NB15	MZ578255	*An. messeae*	M	T	T	C	G	G
NB16	MZ578256	*An. messeae*	M	T	T	C	G	G
NB19	MZ578257	*An. messeae*	M	T	T	C	G	G
NB20	MZ578258	*An. messeae*	M	T	T	C	G	G
NB5	MZ578259	*An. messeae*	M	T	T	C	G	G
T(B)12	MZ578260	*An. daciae*	C	W	W	Y	A	C
T(B)2	MZ578261	*An. daciae*	C	W	W	Y	A	C
T(B)27	MZ578262	*An. daciae*	C	W	W	Y	A	C
T(D)17	MZ578263	Hybrid	C	W	W	Y	R	S
T(D)31	MZ578264	Hybrid	C	W	W	Y	R	S

Table S3. Karyotyping of the specimens based on RFLP analysis of ITS2 sequence.

**Sample ID**	**Species**	**XL**	**2R**	**3R**	**3L**
Teg_20	*An. messeae*	1	11	11	00
Teg_21	*An. messeae*	12	11	01	00
Teg_22	*An. messeae*	11	01	01	00
Teg_23	*An. messeae*	11	11	01	00
Teg_24	*An. daciae*	11	00	00	00
Teg_25	*An. messeae*	1	11	01	00
Teg_26	*An. messeae*	12	11	00	00
Teg_27	*An. daciae*	1	00	00	00
Teg_28	*An. messeae*	11	00	01	00
Teg_29	*An. daciae*	11	00	00	00
Teg_30	*An. messeae*	11	11	11	00
Teg_31	*An. messeae*	11	01	01	00
Teg_32	*An. messeae*	12	11	01	00
Teg_33	*An. messeae*	11	11	01	01
Teg_34	*An. messeae*	11	11	01	11
Teg_35	*An. messeae*	11	11	01	00
Teg_37	*An. daciae*	11	00	00	00
Teg_38	*An. daciae*	11	00	00	00
Teg_39	*An. messeae*	1	11	01	00
Teg_40	*An. messeae*	11	11	01	00

Teg_41	*An. messeae*	11	11	11	00
Teg_42	*An. messeae*	11	01	01	00
Teg_43	*An. daciae*	00	00	01	00
Teg_44	*An. messeae*	2	11	01	00
Teg_45	*An. messeae*	1	11	00	00
Teg_47	*An. messeae*	1	11	01	00
Teg_48	*An. messeae*	11	11	11	00
Teg_49	*An. daciae*	11	00	00	00
Teg_50	*An. messeae*	12	11	00	01
Teg_52	*An. messeae*	1	11	01	01
Teg_53	*An. messeae*	1	11	01	00
Teg_54	*An. messeae*	12	11	00	00
Teg_55	*An. messeae*	11	11	01	00
Teg_57	*An. messeae*	11	11	00	00
Teg_58	*An. messeae*	2	11	01	01
Teg_59	*An. messeae*	12	11	00	00
Teg_60	*An. messeae*	11	11	01	00
Teg_61	*An. messeae*	12	11	11	00
Teg_62	*An. daciae*	01	00	00	00
Teg_63	*An. messeae*	22	11	01	00
Teg_64	*An. daciae*	1	00	00	00
Teg_66	*An. messeae*	11	11	00	00
Teg_67	*An. messeae*	12	11	11	00
Teg_68	*An. messeae*	1	11	00	00
Teg_69	*An. messeae*	1	11	00	00
Teg_70	*An. messeae*	11	11	00	00
Teg_73	*An. messeae*	12	11	00	00
Teg_77	*An. messeae*	11	11	01	00
Teg_78	*An. messeae*	11	11	11	00
Teg_79	*An. messeae*	11	11	00	00
Teg_80	*An. messeae*	1	11	11	00
Teg_81	*An. messeae*	1	11	01	00
Teg_82	*An. messeae*	1	11	11	00
Teg_83	*An. daciae*	01	00	00	00
Teg_84	*An. messeae*	12	11	01	00
Teg_85	*An. messeae*	12	11	11	00
Teg_86	*An. messeae*	1	11	00	00
Teg_87	*An. messeae*	11	11	00	00
