# Supplementary figures and images for: New Cytogenetic Photomap and Molecular Diagnostics for the Cryptic Species of the Malaria Mosquitoes Anopheles messeae and Anopheles daciae from Eurasia
Source: Insects. 2021 Sep 17;12(9):835. doi: 10.3390/insects12090835 (PMC8465136; doi:10.3390/insects12090835)

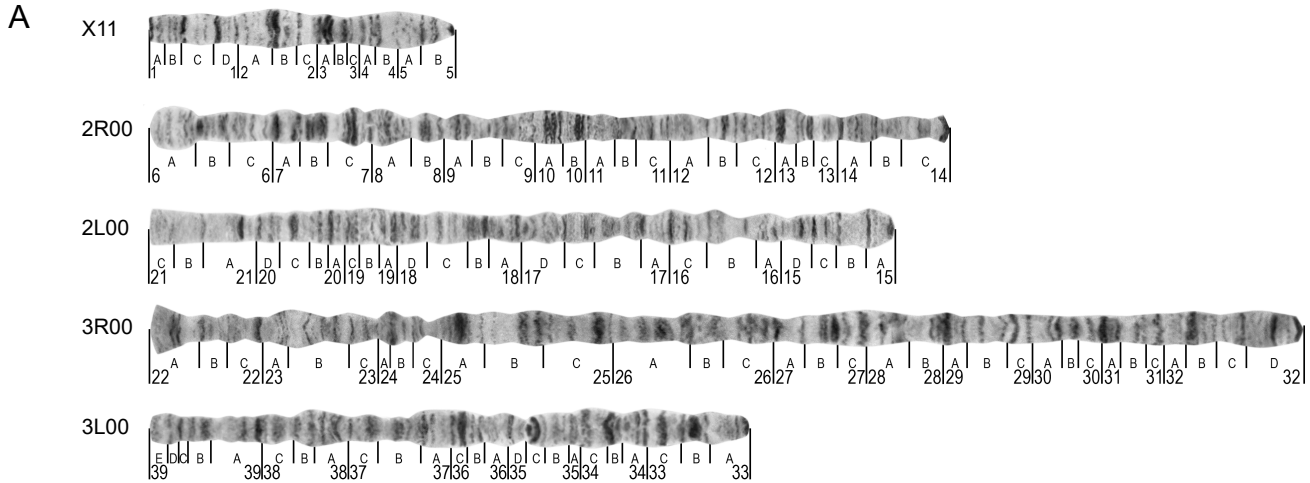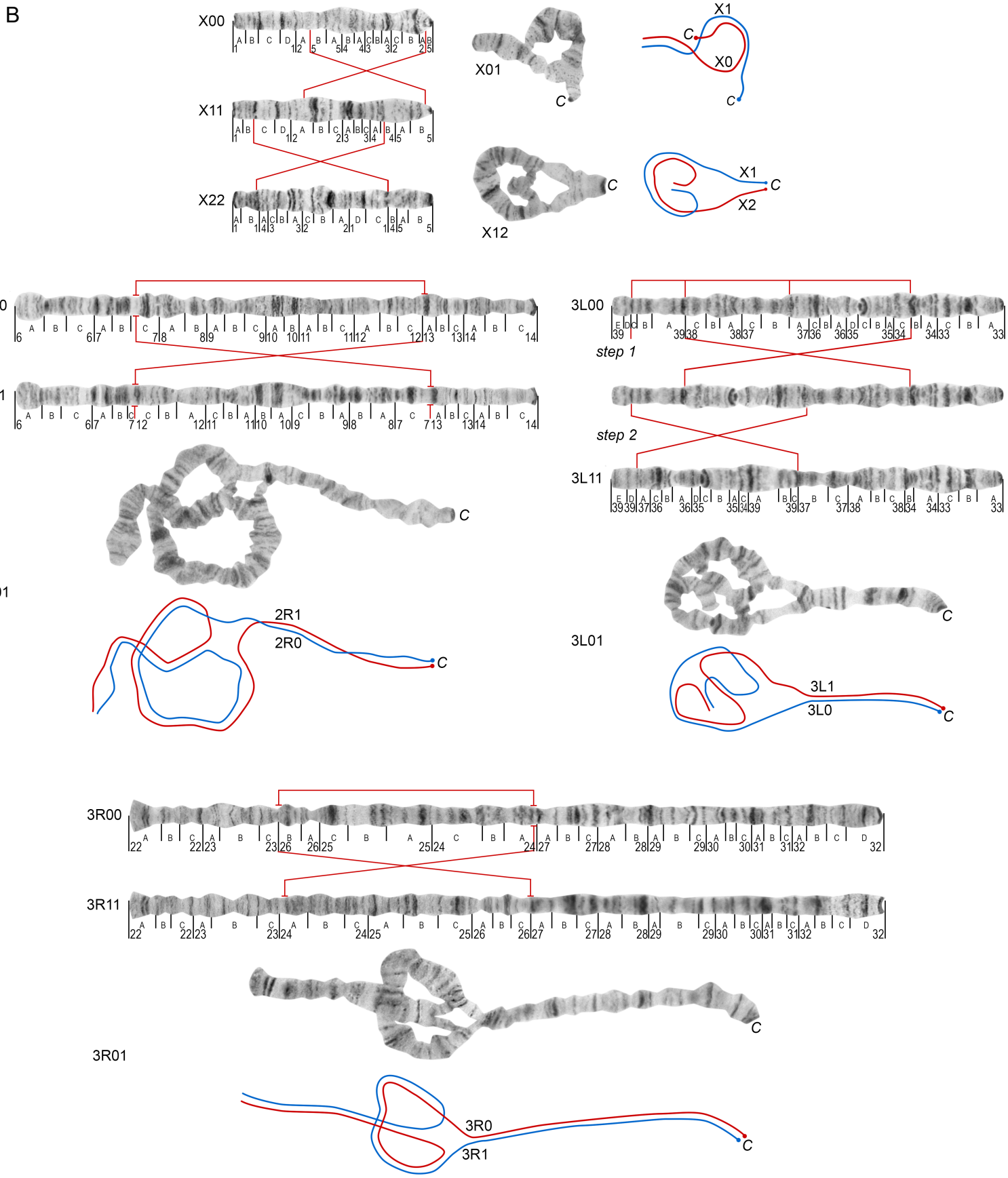

Supplement: Supplementary file 1 [file insects-12-00835-s001.zip › Supplementary figure S1.pdf]
